# Colon‐Targeted Adhesive Hydrogel Microsphere for Regulation of Gut Immunity and Flora

**DOI:** 10.1002/advs.202101619

**Published:** 2021-07-22

**Authors:** Hua Liu, Zhengwei Cai, Fei Wang, Liwen Hong, Lianfu Deng, Jie Zhong, Zhengting Wang, Wenguo Cui

**Affiliations:** ^1^ Department of Gastroenterology Ruijin Hospital Shanghai Jiao Tong University School of Medicine 197 Ruijin 2nd Road Shanghai 200025 P. R. China; ^2^ Department of Orthopaedics Shanghai Key Laboratory for Prevention and Treatment of Bone and Joint Diseases Shanghai Institute of Traumatology and Orthopaedics Ruijin Hospital Shanghai Jiao Tong University School of Medicine 197 Ruijin 2nd Road Shanghai 200025 P. R. China

**Keywords:** colitis, colon‐targeted drug delivery, gut microbiota, hydrogel microsphere, oral administration

## Abstract

Intestinal immune homeostasis and microbiome structure play a critical role in the pathogenesis and progress of inflammatory bowel disease (IBD), whereas IBD treatment remains a challenge as the first‐line drugs show limited therapeutic efficiency and great side effect. In this study, a colon‐targeted adhesive core–shell hydrogel microsphere is designed and fabricated by the ingenious combination of advanced gas‐shearing technology and ionic diffusion method, which can congregate on colon tissue regulating the gut immune‐microbiota microenvironment in IBD treatment. The degradation experiment indicates the anti‐acid and colon‐targeted property of the alginate hydrogel shell, and the in vivo imaging shows the mucoadhesive ability of the thiolated‐hyaluronic acid hydrogel core of the microsphere, which reduces the systematic exposure and prolongs the local drug dwell time. In addition, both in vitro and in vivo study demonstrate that the microsphere significantly reduces the secretion of pro‐inflammatory cytokines, induces specific type 2 macrophage differentiation, and remarkably alleviates colitis in the mice model. Moreover, 16S ribosomal RNA sequencing reveals an optimized gut flora composition, probiotics including *Bifidobacterium* and *Lactobacillus* significantly augment, while the detrimental communities are inhibited, which benefits the intestinal homeostasis. This finding provides an ideal clinical candidate for IBD treatment.

## Graphical abstract

A colon‐targeting and mucoadhesive hydrogel core–shell microsphere is fabricated by advanced gas‐shearing technology for treatment of inflammatory bowel disease. The oral‐administrated microsphere targets the colon where the alginate shell collapses and the HA‐SH‐Ag hydrogel core is released, which congregates on the inflamed colon mucosa for regulation of macrophage differentiation and optimization of gut flora.

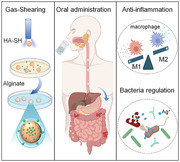

## 1 Introduction

The gut, the largest immune organ in the human body, excessively defends itself from harmless antigens as it is continuously exposed to intestinal contents, which leads to chronic intestinal inflammation.^[^ ^1^, ^2^ ^]^ The recruitment and infiltration of immune cells, the secretion of pro‐inflammatory cytokines, and the accumulation of reactive oxygen species (ROS) cause tissue damage, ulceration, and finally the onset of inflammatory bowel disease (IBD). Bloody diarrhea, abdominal pain, and other complications of IBD could severely impair life quality and lead to an economic burden.^[^ ^3^ ^]^ 5‐aminosalicylic acid (5‐ASA) and glucocorticoids, the first‐line treatment nowadays, however, caused multiple side effects due to their nonspecific anti‐inflammatory property.^[^ ^4^ ^]^ Although novel biotherapies such as infliximab have been developed, these biotherapies are not only expensive but also have a 30% non‐response rate and a 20% increase in resistance rate yearly.^[^ ^5^, ^6^ ^]^ Recently, materials with immune‐regulatory ability have attracted intensive attention.^[^ ^7^, ^8^, ^9^ ^]^ Nanoparticles that target ROS through their redox activity, or cytokines through small interfering RNA‐mediated genetical regulation, and polymers with anti‐inflammatory conjugation were developed to treat colitis.^[^ ^4^, ^7^, ^10^, ^11^ ^]^ Despite the initial success of these immune‐therapies in mice, a healthy flora community also critically matters because without which the immune homeostasis cannot be maintained.

With great abundance and variety, the gut microbiota affects not only homeostasis but also the formation of the immune system. Recently, a microbiome‐wide study has revealed the association between specific microbes and multiple diseases including IBD, tumor, autoimmune diseases, and metabolic diseases, which implied the essential role of gut flora either in IBD or the entire immune system.^[^ ^12^ ^]^ Fecal transplantation is a courageous attempt in gut bacteria regulation, but the complicated mechanism, the lack of clinical standards, and the frequent adverse events have impeded its application. On the other hand, antibiotics appear effective in active IBD, whereas the bacterial resistance and the reduction of probiotics colonies caused by antibiotics offset its benefit.^[^ ^13^, ^14^ ^]^ Moreover, Nguyen et al. have indicated a dose‐dependent positive correlation between antibiotic use and IBD.^[^ ^14^ ^]^ Therefore, there is an urgent need for appropriate interventions to gut microbiome as traditional methods cannot meet expectations.

On the other hand, precise and efficient drug delivery for gut immuno‐microbiota regulation is critical for clinical transformation. Oral administration, characterized by convenience, safety, and its direct effect on local mucosa, is a preferable method of drug delivery in chronic gastrointestinal diseases.^[^ ^15^ ^]^ However, the rapid drug clearance caused by diarrhea,^[^ ^16^ ^]^ the massive drug degradation in digestive juice, and the systematic exposure and absorption diminished the drug bioavailability. While increasing frequency and dosage to maintain the therapeutic effect result in enhanced side‐effect.^[^ ^17^, ^18^ ^]^ Thus, biological material, such as hyaluronic acid (HA), showed therapeutic potential in the lab but failed to reach the expected effectiveness in vivo. Except for the extensive resources and good biocompatibility, HA also exerts an anti‐inflammatory effect by interacting with CD44 on the membrane of immune cells and regulating macrophage differentiation,^[^ ^19^ ^]^ making it an ideal candidate drug for IBD treatment. Nevertheless, its susceptibility to diffusion and degradation in digestive juice limits its application through oral administration.^[^ ^16^, ^20^ ^]^ HA‐based nanoparticles have been developed for various oral‐administrated treatments, but their uncontrollable systemic diffusion reduces the bioavailability and might cause undesirable side effects, which limit their application in IBD treatment.^[^ ^7^, ^21^ ^]^ One way to overcome this dilemma is by applying a colon‐targeted shell that protects the drug from untimely exposure and then collapses to release the drug in a specific location.

**Scheme 1:**
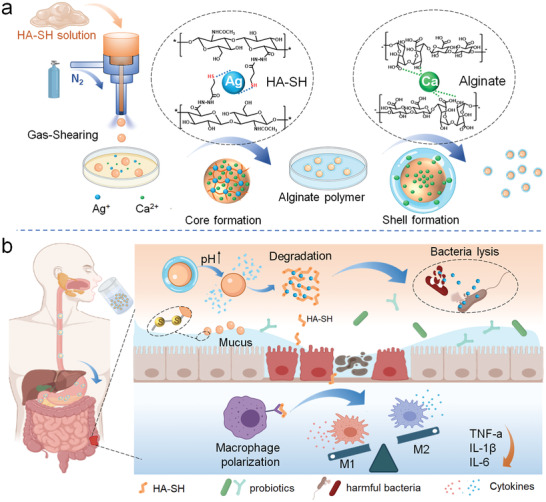
Hydrogel microsphere with core‐shell structure for the colon‐targeted treatment of colitis. a) The gas‐shearing technology was applied to fabricate the HA‐SH‐Ag hydrogel microsphere (HMs) with uniform size, while calcium diffusion from the inside of the microspheres to the surface crosslinks alginate and thus encapsulate core microsphere. b) Oral administrated HA‐SH‐Ag/Alginate‐Ca microspheres (HAMs) target colon to collapse and release HMs. HMs accumulate in inflamed colon mucosa, regulate gut inflammation by suppressing the secretion of pro‐inflammatory cytokines and inducing type 2 (M2) macrophage differentiation dominated immune response, and optimize the composition of gut flora through augmenting probiotics abundance and restraining the detrimental bacterial community.

Based on the significance of intestinal immune response and bacterial structure in the onset of IBD, we innovatively designed a mucoadhesive anti‐inflammatory microsphere with an anti‐acid shell for colon‐targeted delivery, which efficiently regulates gut immune homeostasis and optimizing the composition of flora community through oral administration (**Scheme** **1**). Firstly, silver ion (Ag^+^), a well‐known antimicrobial agent, was utilized to crosslink the anti‐inflammatory thiolated‐HA (HA‐SH), constructing a hydrogel core by using the gas‐shearing technology.^[^ ^22^, ^23^ ^]^ Then, alginate calcium hydrogel, characterized by acid resistance and remarkable ability to crosslink and degrade, was prepared by the ion diffusion method for the colon‐targeted delivery.^[^ ^24^ ^]^ The two‐step fabrication is of high efficiency, precision, and great feasibility in clinical transformation. Besides, the in vitro study revealed the anti‐inflammatory and anti‐bacterial effect of this microsphere, and the in vivo study in mice with dextran sulfate sodium (DSS)‐induced colitis verified the remarkable therapeutic effect of the system. Furthermore, the in vivo colon‐targeted delivery and adhesion were detected through in vivo imaging system (IVIS), and the flora alteration was detected by 16S ribosomal RNA (rRNA) sequencing. Our study proposes a safe and effective drug candidate for clinical IBD treatment.

## 2 Result and Discussion

### 2.1 Synthesis of HA‐SH and Preparation of HA‐SH‐Ag/Alginate‐Ca Microspheres

**Figure 1:**
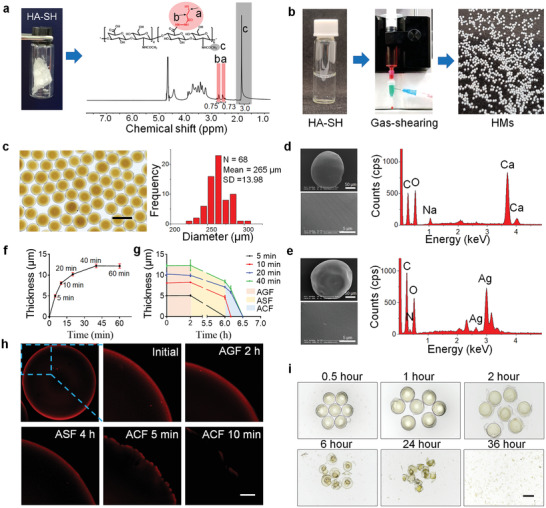
Synthesis of HA‐SH and Preparation of HAMs. a) A photograph (left) and a proton nuclear magnetic resonance spectrum (right) of HA‐SH. The substitution degree of the sulfhydryl group (≈37%) was determined by the integration of the methylene peaks (a and b, red shading) relative to the methyl group of HA (c, grey shading). b) The HA‐SH aqueous solution (left) was fabricated into the HMs (right) by utilizing a gas‐shearing device (middle). c) Optical image of the obtained HMs (top) and the size distribution of the microspheres (bottom). Scale bar 400 µm. d) Representative image of scanning electron microscopy and the elemental analysis of HMs. e) Representative image of scanning electron microscopy and the elemental analysis of HAMs. f) The thickness of the shell that formed by crosslinking alginate with calcium ion (Ca^2+^) changes with the length of dwell time (5, 10, 20, 40, and 60) in sodium alginate solution. g) The changes of shell thickness of microspheres in artificial gastric fluid (AGF, pH 1.0), artificial small intestine fluids (ASF, pH 6.8), and artificial colon fluid (ACF, pH 7.8). h) Representative images of the degradation process of alginate hydrogel shell (red) in AGF, ASF, and ACF successively. Scale bar 20 µm. i) HMs degrade slowly in the presence of hyaluronidase. Scale bar 200 µm.

Inspired by the reaction between thiolated polymers and cysteine‐rich subdomains of the glycoprotein of intestinal mucus,^[^ ^25^, ^26^ ^]^ the thiolated‐HA polymer was prepared according to a previous report (Figure S1, Supporting Information).^[^ ^27^ ^]^ The successful synthesis of HA‐SH was testified by proton nuclear magnetic resonance (^1^H NMR) and Fourier transform infrared spectroscopy (FTIR) (Figure S2, Supporting Information; **Figure** **1**a). The gas‐shearing method was applied to manufacture homogeneous microspheres on a large scale. Superior to the microfluidics method, this method is one‐step and oil‐free, and its production can be scaled up by increasing the flow rate of the polymer solution, which is more effective and biocompatible.^[^ ^23^ ^]^ As shown in Figure 1b,c, the HA‐SH‐Ag hydrogel microspheres (HMs) were fabricated by a gas‐shearing device with a size of low polydispersity (264.64 ± 13.98 µm) and a yield of about 100 mg per minute. Next, HMs were coated with an alginate calcium hydrogel shell as its acid‐resistance can protect the core microspheres for the successful colon‐targeted delivery.^[^ ^24^ ^]^ We visualized the core‐shell structure of the microsphere by labeling alginate and HA‐SH with rhodamine and fluorescein isothiocyanate, respectively (Figure S3, Supporting Information). Under scanning electron microscopy, both HMs and HAMs showed smooth surface, and their element distribution on the surface was detected by energy dispersive spectrometer which further verified the core‐shell structure (Figure 1d,e).

On the other hand, to acquire higher bioavailability, it was essential to make sure that the HAMs are successfully delivered to the colon as well as the rapid release of the core microsphere. Thus, the protective shell with an appropriate thickness which meets the needs of both protection and rapid release of the inner microsphere before and after HAMs reached the colon was required. The hydrogel shell was formed by crosslinking alginate and the Ca^2+^ that diffuses from the inside of the core microsphere to the surface. We observed that the shell thickened (ranging from 0 µm to 12 µm) as the dwell time (ranging from 5 to 40 min) of HMs in alginate aqueous solution increased (Figure 1f). The thickness stopped increasing as the dwell time reached 40 min as a consequence of the exhaustion of Ca^2+^. Next, we successively immersed the HAMs with different shell thicknesses in artificial digestive fluids which simulate pH value in different sections of the digestive tract. It turned out that a dwell time of 10 min, in another word, a thickness of about 8 µm, best meets our requirement (Figure 1g). The microsphere with an 8 µm hydrogel shell remained relatively intact in both artificial gastric fluid and artificial small intestine fluid, but degraded within 10 min in an artificial colon fluid (Figure 1h). Also, to verify the well‐designed degradation of the shell in vivo, we searched and collected the HAMs in the digestive tract of mice at different time points, and the collected microspheres were observed under a confocal microscope. As is shown in Figure S4, Supporting Information, the microspheres collected from the stomach and small intestine showed relatively intact shell under a confocal microscope, while the microsphere from the colon was almost invisible, which verified the colon‐targeted delivery of HAMs. After the disintegration of shell hydrogel, we observed that the core hydrogel of HAMs swelled and degraded gradually within 1 to 2 days in the artificial colon fluid containing hyaluronidase (Figure 1i), which is conducive to the sustained drug release in vivo.

### 2.2 Characteristics of HAMs

**Figure 2:**
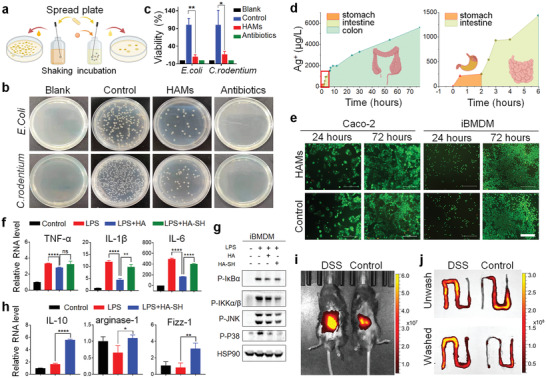
Characteristics of HAMs. a) Schematic illustration of the anti‐bacterial experiment. b) The anti‐bacterial activity of HAMs was shown by the spread plate method. c) The bacterial viability of *Escherichia coli* (*E. coli*) and *Citrobacter rodentium* (*C. rodentium*) in (b) were measured by colony count. The experiment was repeated three times. d) The silver ion (Ag^+^) release from 100 mg HAMs in 1 mL artificial gastric, intestinal, and colon fluids was detected by inductively coupled plasma mass spectrometer. e) The cytotoxicity of HAMs was examined in Caco‐2 and immortalized bone marrow‐derived macrophage (iBMDM) by using the calcein acetoxymethyl ester/propidium iodide cell viability kit. Scale bar 500 µm. f) mRNA expression of the cytokines including interleukin‐6 (IL‐6), interleukin‐1*β* (IL‐1*β*), and tumor necrosis factor‐*α* (TNF‐*α*). g) Western blot analysis of protein level of phosphorylated inhibitor of nuclear factor *κ*B (P‐I*κ*B*α*) and phosphorylated inhibitory kappa B kinase *α*/*β* (P‐IKK*α*/*β*), phosphorylated‐P38 and phosphorylated c‐Jun N‐terminal kinase (P‐JNK) in iBMDM with different treatment. h) mRNA levels of M2 macrophage‐related genes, including interleukin‐10 (IL‐10), arginase‐1, and found in inflammatory zone‐1 (Fizz‐1). i,j) Healthy mice and mice with dextran sulfate sodium (DSS)‐induced colitis were imaged by in vivo imaging system after the oral gavage of indocyanine green (ICG)‐HAMs, and the intestine with strong signals was removed and then imaged before and after washing. The experiments were repeated three times. Significance between every two groups was assessed by using Mann–Whitney U‐test. ns, not significant; * *p* < 0.05, ** *p* < 0.01, **** *p* < 0.0001.

Ag^+^ and HA‐SH polymers were gradually released during the degradation process of HAMs. Previous studies reported that Ag^+^ exerts its ant‐bacterial ability by damaging cell membranes and affect iron‐sulfur proteins to reduced their enzymatic activity.^[^ ^28^ ^]^ Ag^+^ has been used as an antimicrobial agent from time immemorial,^[^ ^22^ ^]^ while there is considerable evidence supporting the vital role of abnormal flora community in the onset of IBD. Thus, we first evaluated the antibacterial ability of HAMs by the spread plate method (**Figure** **2**a) and the Kindy–Bauer method.^[^ ^29^ ^]^ The significantly reduced bacterial colony counting (Figure 2b,c) and inhibitory zone diameter (Figure S5, Supporting Information) in the HAMs group indicated the good anti‐bacterial ability of HAMs against *Escherichia coli and Citrobacter rodentium*, which is a pathogen that can induce mice colitis, yet relatively weaker than antibiotics. On the other hand, it is well‐known that Ag^+^ is cytotoxic, but it is also a misconception without consideration of dosage and practical application. It was reported that Ag^+^ of a slight trace is more toxic to microorganisms than to human beings according to the literature.^[^ ^22^, ^30^ ^]^ Due to the complexity and fickleness of the intestinal environment, it was difficult to evaluate the in vivo concentration of Ag^+^. Therefore, we only measured the in vitro Ag^+^ release, and evaluated its toxicity by co‐culturing HMs with intestinal epithelial cell line Caco‐2 and macrophage cell line immortalized bone marrow‐derived macrophage (iBMDM) and then testing the cell viability. As shown in Figure 2d,e, and Figure S6, Supporting Information, the released Ag^+^ during the degradation of HAMs was infinitesimal, and both cells showed high viabilities, which indicated the satisfactory biocompatibility of HAMs in vitro, but the in vivo verification of HAMs safety also needs to be conducted.

In addition, the anti‐inflammatory ability of the thiol‐modified HA was estimated in vitro by testing the mRNA expression and secretion of cytokines stimulated by lipopolysaccharide (LPS) in the iBMDM cell line. As expected, HA‐SH, compared to HA, showed superior anti‐inflammatory ability by inhibiting the mRNA expression as well as the secretion of interleukin‐6 (IL‐6), interleukin‐1*β* (IL‐1*β*), and tumor necrosis factor‐*α* (TNF‐*α*) (Figure 2f; Figure S7, Supporting Information). Next, we tried to dig the upstream signaling pathway that mediates the anti‐inflammatory property of HA‐SH; we found that the protein level of phosphorylated inhibitor of nuclear factor *κ*B (P‐I*κ*B*α*) and phosphorylated inhibitory kappa B kinase *α*/*β* (P‐IKK*α*/*β*), P‐P38, and phosphorylated c‐Jun N‐terminal kinase (P‐JNK) were downregulated in HA‐SH treated iBMDM (Figure 2g). These phosphorylated proteins are key molecules of nuclear factor *κ*B (NF‐*κ*B) and mitogen‐activated protein kinase (MAPK) signaling pathway, the main inflammatory signaling pathway in the human body.^[^ ^31^, ^32^ ^]^ Moreover, the inhibition of NF‐*κ*B and MAPK pathways in the HA‐SH treated group is much more significant than HA treated group. According to the literature, the thiol group is capable of converting the oxidized form of glutathione to reduced form, and the reduced glutathione can mediate the inhibition of protein tyrosine phosphatase (PTP).^[^ ^33^, ^34^, ^35^ ^]^ Some research showed that the deletion of macrophage‐specific PTP‐1B can markedly reduce inflammation and protect mice against LPS‐induced endotoxemia.^[^ ^36^, ^37^ ^]^ This evidence suggests that HA‐SH may rely on its thiol group to inhibit PTP activity so as to promote its anti‐inflammatory ability. On the other hand, type 1 macrophage (M1) accumulates and contributes to the secretion of the above cytokines in colon tissue of IBD patients according to the literature,^[^ ^2^, ^38^ ^]^ while type 2 macrophage (M2), in contrast, exerts anti‐inflammatory and wound‐healing ability.^[^ ^39^ ^]^ Thus, to elucidate the anti‐inflammatory mechanism of HA‐SH, we conducted quantitative real‐time PCR (qRT‐PCR) to evaluate the relative mRNA expression of M2‐related genes, and found that HA‐SH significantly upregulated mRNA level of interleukin‐10 (IL‐10), arginase‐1, and found in inflammatory zone‐1 (Fizz‐1) in iBMDM cell (Figure 2h), which suggested that HA‐SH exerts its anti‐inflammation ability by inducing the M2 differentiation. Additionally, the experiment on the animal model was needed to verify the in vivo therapeutic effect.

On the other hand, the combination of colon‐targeted drug delivery and mucoadhesive property could maximize bioavailability. We mentioned previously that the remaining thiol groups of HA‐SH‐Ag hydrogel could form disulfide bonds with mucin on the surface of colon mucosa after the collapse of shell hydrogel, which prolongs the dwell time of the microsphere.^[^ ^26^ ^]^ Moreover, HA‐SH also possesses a negative charge due to its abundant carboxyl group, which helps its retain on the inflamed colon tissue with positively charged proteins such as transferrin and antimicrobial peptides.^[^ ^40^ ^]^ To verify the mucoadhesive property in vivo, the mice were orally administrated with HAMs which were fabricated by indocyanine green (ICG)‐modified HA‐SH, and were observed under IVIS. The stronger fluorescence signal in mice with DSS‐induced colitis in Figure 2i suggested that the ICG‐HAMs are more concentrated in this group. The alteration of fluorescence intensity before and after PBS washing between intestine with or without inflammation implies that the dwell time of HAMs is prolonged in mice with DSS‐induced colitis (Figure 2j), which verified the mucoadhesive property of HMs in vivo.

### 2.3 Excellent Therapeutic Efficacy of HAMs in the Mice Model with DSS‐Induced Colitis

**Figure 3:**
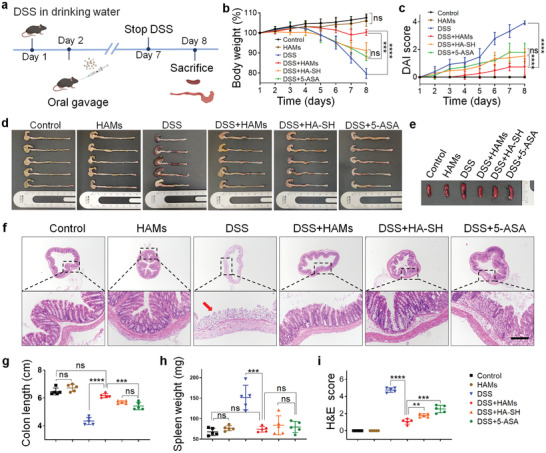
Excellent therapeutic efficacy of HAMs in the mice model with DSS‐induced colitis. a) Experimental design. The mice were provided with sterile water or water containing 3% DSS for 7 days. Oral administration of treatments was given from the second day. All mice were sacrificed two days after DSS drinking stopped. b) Daily changes of body weight were recorded in detail and analyzed, *n* = 5. c) Everyday disease activity index (DAI) scores were calculated and analyzed, *n* = 5. d) Macroscopic colon appearance of each group was shown, *n* = 5 per group. e) Representative macroscopic spleen appearance of each group. f) Representative hematoxylin and eosin (H&E) staining images of colon tissue of each group. Scale bar is 200 µm. g) Colon length was measured and analyzed, *n* = 5. h) Spleen weight was measured and analyzed, *n* = 5. i) Colonic damage scores according to H&E staining were analyzed in each group. Significance between every two group was assessed by using Mann–Whitney U‐test; ns, not significant; * *p* < 0.05, ** *p* < 0.01, *** *p* < 0.001, **** *p* < 0.0001.

The above research investigated the anti‐inflammatory ability of HA‐SH and the anti‐bacterial potential in vitro, as well as the mucoadhesive capacity of HAMs in the inflamed colon mucosa. Thus, it is imperative to carry out the in vivo study to evaluate the therapeutic effect of HAMs. DSS‐induced colitis, one of the most commonly used IBD animal models, was introduced to simulate IBD.^[^ ^41^ ^]^ Oral administration of DSS via drinking water can damage epithelial cells, which breaks the intestinal barrier and cause the subsequent incursion of luminal microbiota that result in inflammation.^[^ ^42^ ^]^ We chose 5‐aminosalicylic acid (5‐ASA), one of the first‐line anti‐inflammatory treatments of IBD, as the positive control to evaluate the therapeutic effect of HAMs.^[^ ^1^, ^16^ ^]^ All mice were randomly divided into six groups, including the control group, the HAMs group, the DSS group, the DSS + HAMs group, the DSS + HA‐SH group, and the DSS + 5‐ASA group. The first two groups were provided with sterile water, while the rest with water containing 3% w/v DSS. Other treatments were given separately by oral gavage every day (**Figure** **3**a).

In comparison with the DSS group, the DSS + HAMs group showed larger bodyweight (*p* < 0.0001, Figure 3b), lower disease activity index (DAI) (Figure 3c; Figure S8, Supporting Information), longer colon length (*p* < 0.0001, Figure 3d,e), and smaller spleen weight (*p* < 0.001, Figure 3f,g), all of which indicated that HAMs prominently protected the mice from DSS‐induced colitis. According to the colon damage score from the hematoxylin‐eosin (H&E) staining in Figure 3h,i (*p* < 0.001), treatment of HAMs also helped maintain the integrity of colon epithelium and lessen the infiltration of pro‐inflammatory cells of the mucosa. The evidence shown in mice demonstrated that the HAMs is a promising therapy. Moreover, we also compared the therapeutic effect between HAMs and 5‐ASA, as 5‐ASA is the first‐line drug in IBD treatment but with a risk of relapse and multiple adverse effects.^[^ ^1^, ^43^ ^]^ It was observed that HAMs performed much better than 5‐ASA in colitis alleviation in various aspects, including body weight, colon length, colon damage score, and neutrophil infiltration, while the oral administration of HA‐SH solution showed a similar therapeutic effect in comparison with 5‐ASA (Figure 3). The distinction of therapeutic effect between HAMs and HA‐SH might arise from the drug depletion in the delivery process or the lack of Ag^+^ released.

### 2.4 HAMs Inhibit Intestinal Inflammation and Prompt Tissue Repair

**Figure 4:**
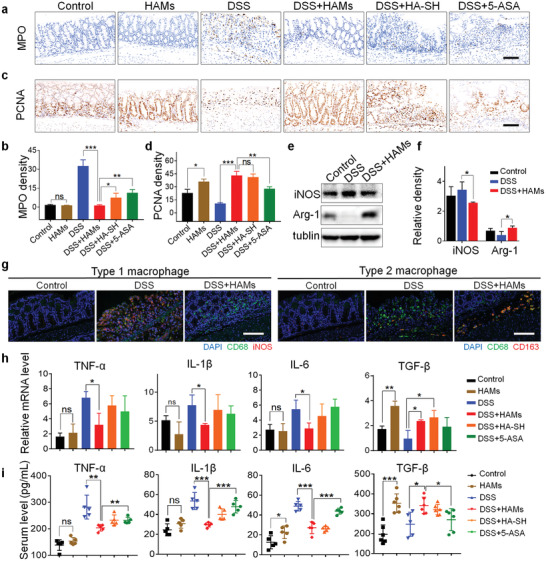
HAMs inhibit intestinal inflammation and prompt tissue repair. a) Representative immunohistochemical staining of myeloperoxidase (MPO); scale bar is 100 µm. b) The semi‐quantitative analysis of immunohistochemistry staining of MPO. c) Representative immunohistochemical staining of proliferating cell nuclear antigen (PCNA); scale bar is 100 µm. d) The semi‐quantitative analysis of immunohistochemistry staining of PCNA. e) Representative image of western blot analysis of inducible nitric oxide synthase (iNOS) and arginase‐1 (Arg‐1) level in mice colon tissue. f) Relative iNOS and Arg‐1 levels were quantified by ImageJ software. g) Immunofluorescence analysis of type 1 macrophage (iNOS red, CD68 green, and 4′,6‐diamidino‐2‐phenylindole [DAPI] blue) and type 2 macrophage (CD163 red, CD68 green, and DAPI blue) in colon tissue visualized under confocal microscopy' scale bar is 100 µm. h) Colonic mRNA levels of TNF‐*α*, IL‐1*β*, IL‐6, and transforming growth factor‐*β* (TGF‐*β*). i) The serum concentration of IL‐6, IL‐1*β*, TNF‐*α*, and TGF‐*β*. Significance between every two groups was assessed by using Mann–Whitney U‐test. ns, not significant; * *p* < 0.05, ** *p* < 0.01, *** *p* < 0.001.

We then conducted an in‐depth analysis to figure out how HAMs protect the mice from DSS‐induced colitis. The proportion of myeloperoxidase (MPO)‐positive neutrophils significantly augmented in the colon tissue of the DSS group, while HAMs remarkably reduced its congregation in colon tissue, further verifying the anti‐inflammatory capability of HAMs (*p* < 0.001, **Figure** **4**a,b). Interestingly, we also observed that, with the administration of HAMs, the epithelium was relatively intact under DSS treatment. Moreover, a much higher expression of proliferating cell nuclear antigen (PCNA), a crucial tissue marker of cell proliferation, was observed in the groups with the administration of HAMs or HA‐SH (*p* < 0.001, Figure 4c,d), implying that HA‐SH may exert an ability of tissue repairing as HA can do in a previous report.^[^ ^44^ ^]^ Besides, the previous in vitro study shows that HA‐SH regulated the differentiation of macrophages in the iBMDM cell line. Hence, we tested the protein level of inducible nitric oxide synthase (iNOS) which is the M1 marker, and arginase‐1, the M2 marker, in colon tissue by western blot. We found that administration of HAMs significantly up‐regulated the arginase‐1 and down‐regulated the iNOS expression instead (Figure 4e,f). Also, immunofluorescence analysis of macrophages in colon tissue suggested that HAMs augmented the differentiation of M2 macrophage and suppressed the M1 in DSS‐induced colitis (Figure 4g). Besides, we detected the colonic mRNA expression and serum concentration of the tissue repair‐associated cytokine‐transforming growth factor‐*β* (TGF‐*β*) and pro‐inflammatory cytokines, including IL‐6, IL‐1*β*, and TNF‐*α*. As expected, both the expression and secretion of pro‐inflammatory cytokines were held down, while TGF‐*β* increased under HAMs treatment (Figure 4h,i), which is consistent with our previous finding in vitro. Except for treatment efficacy, we also assessed the drug safety in vivo. The H&E staining of the major organs tissue including heart, lung, liver, and kidney in mice with HAMs treatment was normal and similar to other groups, suggesting that the good biological compatibility of HAMs and that the cytotoxic effect of Ag^+^ is negligible in vivo (Figure S9, Supporting Information).

### 2.5 16S rRNA Sequencing Analysis of Gut Microbiota Regulated by HAMs

**Figure 5:**
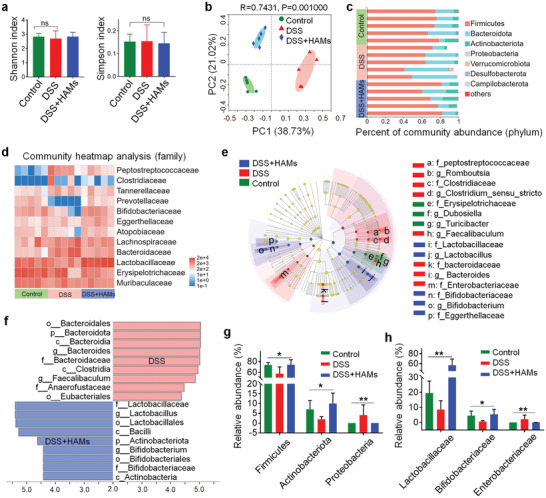
16S ribosomal RNA (rRNA) sequencing analysis of gut microbiota regulated by HAMs. a) Shannon and Simpson index of observed operational taxonomic units showed the *α*‐diversity of the microbial community. b) Principal co‐ordinates analysis showed the *β*‐diversity of the gut microbiome. Each point represents each mouse and *n* = 5 for each group. The significance of clustering was determined using analysis of similarities (ANOSIM). c) Community histogram showed the microbial compositional profiling at the phylum level. Each row represents each mouse and *n* = 5 for each group. d) Heatmap exhibited the relative abundance of microbial compositional profiling at a family level. Each column represents each mouse and *n* = 5 for each group. e) A cladogram showed the difference in richness and the group with a significant difference in abundance. The brightness of each dot is proportional to its effect. f) Linear discriminant analysis (LDA) identifies the significantly abundant genus in different groups. Taxa that meeting an LDA significant threshold of 4 are shown. g) Relative abundance of microbiota that is significantly altered at the phylum level. The Kruskal–Wallis test was utilized for statistical analysis. h) Relative abundance of microbiota that is significantly altered at the family level. The Kruskal–Wallis test was applied for statistical analysis. ns, not significant; * *p* < 0.05, ** *p* < 0.01.

It is well‐known that the perturbation of intestinal flora is closely associated with IBD, and that Ag^+^ has a bactericidal effect in a broad spectrum.^[^ ^28^, ^45^ ^]^ However, wiping out specific colonies such as probiotics might also harm the health of the gut microenvironment. Thus, to investigate if and how HAMs affect the composition or abundance of gut microbiota, we carried out the in‐depth analysis of mice enterobacterial composition by applying the advanced 16S rRNA sequencing technology. We found no significant distinction in both community richness and alpha‐diversity between the three groups (the control group, the DSS group, and the DSS + HAMs group) (**Figure** **5**a; Figure S10, Supporting Information), which is probably due to the short modeling period. However, principal co‐ordinates analysis indicated that the treatment of HAMs significantly altered the composition of microflora in mice with colitis (Figure 5b). Here, DSS treatment also changed the composition of the bacteria community in comparison with the control group; we reviewed the literature and found that it is a common phenomenon.^[^ ^7^, ^46^, ^47^ ^]^ It is understandable because DSS may somehow affect the bacteria community, and on the other hand, colitis is always accompanied by microbiota alteration.^[^ ^48^ ^]^ The general enterobacteria composition of each sample at the phylum and family level (Figure 5c,d) was shown by bar chart and heatmap, respectively. Linear discriminant analysis (LDA) effect size was also analyzed to find out the taxa which are differentially abundant in these groups, and the LDA score showed the dominant groups and their impact at various taxonomic levels from phylum to genus (Figure 5e,f).

The administration of HAMs helped maintain a similar microbiota composition at the phylum level compared with the control group. While the *Firmicutes* and *Actinomycetes* abundance was found decreased, and the *Proteobacteria* abundance was found raised in the DSS group (Figure 5g), which were consistent with the flora composition in IBD patients reported in the literature.^[^ ^45^ ^]^ Furthermore, *Lactobacillaceae* and *Bifidobacteriaceae*, the probiotics that benefit the health of intestinal microenvironment by competitive inhibition of harmful microbes and regulating intestinal immune response,^[^ ^49^ ^]^ were found higher in abundance in the group with HAMs treatment. In contrast, the abundance of *Enterobacteriaceae* which usually accumulates in the colon of IBD patients was found reduced (Figure 5h). The change of abundance among those bacteria indicated the selective antimicrobial property of HAMs, which is in accord with a previous report about the bactericidal selectivity of Ag^+^.^[^ ^22^ ^]^ These results indicated that HAMs could optimize the composition of gut microbiota in mice. Therefore, HAMs could be a good drug candidate in treating active colitis by reducing the side effects of antibiotics or preventing severe infection from the drug‐resistant bacteria.

## 3 Conclusions

In summary, we designed a novel therapeutic system—an orally administrated core–shell microsphere that targets the colon to collapse and release HA‐SH‐Ag hydrogel to regulate intestinal inflammation and flora composition, as well as the tissue repair process. Owing to the protective alginate hydrogel shell that targets the colon and the mucoadhesive property of HA‐SH that makes the microspheres aggregate to the inflamed colon mucosa, this system reduced the systematic exposure and prolonged the local drug dwell time, which maximized the drug bioavailability. The core microsphere not only regulates the gut inflammation through suppressing the secretion of pro‐inflammatory cytokines and inducing the M2 differentiation‐dominated immune response, but it also plays a role in positive flora regulation through augmenting probiotics abundance and restraining the detrimental bacterial community. Moreover, the fabrication process combined with the advanced gas‐shearing technology and ionic diffusion method is highly efficient and has great feasibility in clinical transformation, providing a versatile platform for oral delivery of drugs or cells. Also, we revealed the neglected superior anti‐inflammatory property of HA‐SH. In general, this therapeutic system provides new insight into the comprehensive IBD treatment.

## 4 Experimental Section

### Synthesis of HA‐SH

HA‐SH was prepared by the condensation reaction with dimethoxy‐1,3,5‐triazine‐2‐yl‐4‐methylmorpholine between hyaluronic acid (360 kDa, Fruida, Shandong, China) and 3,3′‐Dithiobis‐(propanoic dihydrazide), and the reduction of disulfide bond by adding tris(2‐carboxyethyl) phosphine hydrochloride, as previously reported.^[^ ^27^ ^]^

### Preparation of HAMs

The core microsphere was fabricated by using a gas‐shearing device. The aqueous solution of HA‐SH (4% w/v) was injected through a coaxial needle and was cut into homogeneous droplets by the shearing force generated by nitrogen flow (0.4 L min^−1^). The collecting bath contains calcium nitrate (Ca (NO_3_)_2_, 0.1 mol L^−1^) and silver nitrate (AgNO_3_, 0.2 mol L^−1^) aqueous solution, the latter of which provided the Ag^+^ for crosslinking HA‐SH to form HA‐SH‐Ag hydrogel microspheres. The core microspheres were collected and washed twice to remove the residual ions on the surface. It was then added to the sodium alginate solution, as the calcium ions within the microsphere will disperse to the surface and crosslink sodium alginate forming a protective shell on the surface. The whole operation was carried out in a sterile environment. Finally, the microspheres with core–shell structure were collected and stored at 4 °C and avoiding light.

### Characterization of the HA‐SH and the Microsphere

Both Fourier transform infrared spectrometer (Nicolet 6700) and 600 MHz ^1^H NMR Spectrometer (Bruker Avance spectrometer) were applied to verify the material structure. The core–shell structure of the microsphere was observed under a confocal microscope (Carl Zeiss, Germany). The microspheres were then freeze‐dried and observed by using the field‐emission scanning electron microscopy (FEI, Sirion 200), while the microelement composition of the microsphere and the planar distribution was measured by the energy dispersive spectrometer (Oxford).

### Shell thickness Test and Degradation Assay

The core microsphere was immersed in sodium alginate solution (1% w/v) for 5, 10, 20, 40, and 60, respectively, and the shell thickness was evaluated by using the confocal microscope to find out how the shell thickness change according to the soaking time. Then, the microspheres with different shell thicknesses were immersed in the artificial gastric fluid (2 h), artificial small intestine fluid (4 h), and artificial colon fluid, respectively. The shell thickness was monitored at different time points to find the appropriate shell thickness for the precise drug delivery. To research the degradation process of the core microsphere, it was immersed in artificial colon fluid containing 5% w/v hyaluronidase, and the degradation process of HA‐SH‐Ag hydrogel was observed under an optical microscope at different time points (30 min, 1 h, 2 h, 6 h, 24 h, 36 h).

### In Vitro Antibacterial Assay

The antibacterial ability of HAMs was evaluated by using *E. coli*, an opportunistic pathogen, and *C. rodentium*, a pathogen that induces colitis in mice. 1) Spread plate counting method: *E. coli* and *C. rodentium* were grown in Luria‐Bertani (LB) medium separately, and the microspheres and penicillin–streptomycin solution were added separately into the different groups. All cultures were cultivated in a shaking incubator at 37 °C for 24 h and then diluted for spread plate (LB‐ager medium). After incubating at 37 °C for 12 h, the colonies on the plate were counted to analyze the antibacterial ability of the microsphere. 2) Kirby–Bauer Method: *E. coli* and *C. rodentium* were grown in LB medium for 24 h before use. Then, the microbial cultures were diluted to 0.5 McFarland's standard and were swabbed evenly on the plate. Next, the 6 mm round filter paper containing 20 uL microsphere extract was adhered to the plate. Zones of inhibition were photographed and measured after 24 h of incubation at 37 °C. The penicillin–streptomycin solution was applied as a positive control. The experiment was repeated at least three times.

### Ag^+^ Release

The release amount of Ag^+^ was detected by using PBS solutions with different pH values. 100 mg microspheres were immersed in 1 mL artificial gastric fluids (pH = 1) for 2 h, artificial intestine fluids (pH = 6.8) for 4 h, and artificial intestine fluids (pH = 7.8) for 72 h, successively. 500 µL of the solution was extracted and supplemented with equivoluminal fluid at a certain time interval. Finally, an inductively coupled plasma mass spectrometer (ICP‐MS, Thermo Fisher) was applied to quantify the release of Ag^+^ in the solution.

### Cell Culture

Mice iBMDM and human colon carcinoma cell line Caco‐2 (ATCC; USA), were cultured in the 1640 medium and the Dulbecco's Modified Eagle's medium (GIBCO) respectively, and the mediums were supplemented with 10% fetal bovine serum (GIBCO).

### Biocompatibility Evaluation

Cell counting kit‐8 (CCK‐8) assay (Beyotime, China) and calcein acetoxymethyl ester/propidium iodide (Calcein AM/PI) cell viability/cytotoxicity assay kit (Beyotime) were utilized to test the biocompatibility of the HA‐SH‐Ag hydrogel microsphere. Cells were co‐cultured with the microspheres for 3 days. The cell viability was tested by Calcein AM/PI cell viability/cytotoxicity assay kit and CCK‐8. After incubating with the Calcein AM/PI buffer for 30 min, the live cell (green) or dead cell (red) were observed under a fluorescent microscope. By incubating the CCK‐8 reagent with the cells for 2 h, the cell viability was detected by measuring the absorbance in 450 nm using a Microplate Reader.

### Assessment of the Anti‐Inflammatory Effect

iBMDM cells were seeded into 6‐well plates; the cells were stimulated by LPS (1 µg mL^−1^) and then incubated with 100 µL of 4% w/v aqueous solution of HA‐SH or HA to test the anti‐inflammatory effect of HA‐SH. Total RNA of the cells and the colon tissue was extracted by using Trizol reagent (Takara, Japan), and then 1 µg RNA was transcribed into cDNA by Hifair II 1st Strand cDNA Synthesis Kit (Yeasen, China). Next, qRT‐PCR was conducted by utilizing the Hieff qPCR SYBR Green Master Mix (Yeasen) on LightCycler 480 Instrument II (Roche). The relative mRNA expression of the following genes was normalized by using beta‐actin as the endogenous control. The primer sequences used were as follows:

Beta‐actin: Forward CGTTGACATCCGTAAAGACC
Reverse TAGGAGCCAGAGCAGTAATC
arginase‐1: Forward CTCCAAGCCAAAGTCCTTAGAG
Reverse GGAGCTGTCATTAGGGACATCA
Interleukin 10: Forward CAGGGATCTTAGCTAACGGAAA
Reverse GCTCAGTGAATAAATAGAATGGGAAC
Fizz‐1: Forward CCAATCCAGCTAACTATCCCTCC
Reverse ACCCAGTAGCAGTCATCCCA
TNF‐*α*: Forward CAGGCGGTGCCTATGTCTC
Reverse CGATCACCCCGAAGTTCAGTAG
IL‐1*β*: Forward TTCAGGCAGGCAGTATCACTC
Reverse GAAGGTCCACGGGAAAGACAC
IL‐6: Forward CTGCAAGAGACTTCCATCCAG
Reverse AGTGGTATAGACAGGTCTGTTGG
TGF‐*β*: Forward CCAGATCCTGTCCAAACTAAGG
Reverse CTCTTTAGCATAGTAGTCCGCT

### In Vivo IVIS Imaging

Healthy mice and mice with DSS‐induced colitis were fasted for 24 h, and their abdominal hair was all shaved before imaging. The mice were given 20 mg of ICG‐HAMs by oral gavage. Four hours later, the mice were imaging under IVIS. The intestines with strong signals were removed. They were imaged by IVIS before and after irrigation of 10 mL PBS with a syringe to measure the mucoadhesive property of HAMs.

### Animals and DSS‐Induced Mice Model

C57BL/6 mice (male, 8 weeks) were obtained from the Animal Center of Shanghai Jiao Tong University School of Medicine, and the Animal Care Committees of Shanghai Jiao Tong University School of Medicine approved the experimental protocols. All mice were housed in a specific‐pathogen‐free (SPF) environment. The mice were randomly divided into five groups. Acute colitis was induced by 7 days of water‐mediated administration of DSS (Millipore). The first two groups received sterile water without DSS, while the rest received DSS solution (2.5% w/v) which was prepared fresh every 2 days. During this period, stool consistency, fecal blood, and weight loss of mice were all daily recorded to determine the DAI.^[^ ^6^ ^]^ Two days after the administration of DSS stopped, all mice were euthanized and the colon, spleen, liver, lung, and heart were collected for further analysis.

### H&E Staining, Immunohistochemistry, and Immunofluorescence Staining

The tissue samples were fixed in 4% paraformaldehyde. After the dehydration in ethanol, the tissue was embedded in paraffin then sectioned with a thickness of 4 µm. The histopathological feature was tested via H&E staining. The tissue damage score was evaluated according to the previous report.^[^ ^42^ ^]^ To perform immunohistochemistry and immunofluorescence staining, the tissue sections were deparaffinized, rehydrated, and rinsed, followed by antigen retrieval (heat‐induced epitope repair method) and blocking (goat serum). Next, the tissue sections were incubated with primary antibody overnight and followed by the biotinylated secondary antibodies (for immunohistochemistry staining) or Alexa Fluor conjugated secondary antibody (for immunofluorescence staining). The DAB Horseradish Peroxidase Color Development Kit (Dako, Agilent Technologies, USA) was applied for color reaction in immunohistochemistry staining. Finally, the tissue sections were observed under the optical microscope or fluorescence microscope. The following primary antibodies were used: P‐I*κ*B*α* (Proteintech, 1:1000), P‐IKK*α*/*β* (Proteintech, 1:1000), P‐P38 (CST, 1:1000), P‐JNK (CST, 1:1000), heat‐shock protein 90 (CST, 1:3000), MPO (CST, 1:500), PCNA (CST, 1:500), CD68 (Abcam, 1:500), CD163 (Abcam, 1:5000), arginase‐1 (Proteintech, 1:1000), iNOS (Proteintech, 1:1000), Tubulin (CST, 1:5000). The Image J software was applied for scoring the immunohistochemistry picture.

### Serum and Cell Culture Supernatant ELISA Analysis

Mice blood was centrifuged for 15 min at 3000 rpm to acquire the serum. Cell culture medium was collected and centrifuged for 5 min at 400 g for supernatant. The concentration of IL‐6, IL‐1*β*, TNF‐*α*, as well as TGF‐*β* in mice blood and cell culture supernatant was measured using the Enzyme‐linked Immunosorbent Assay Kit (MEIMIAN, China). The samples were transferred to an ELISA plate with relevant antibody, after incubating for 2 h, the plate was washed five times with washing buffer. Then, the secondary antibody was added to the plate. After incubating for 60 min, the plate was washed five times. Next, the HRP‐conjugate reagent was added and incubated for 30 min. After the washing process (five times), 3,3″,5,5″‐tetramethylbenzidine substrate solution (15 min) and stop solution were added successively. Finally, the cytokine concentration was measured according to the absorbance at 450 nm by using a Microplate Reader.

### Microbiome Analysis

Feces of each mouse were collected separately on day 8 and stored at −20 °C. Total DNA was extracted by using the E.Z.N.A. soil DNA Kit (Omega Bio‐Tek, Norcross, GA, USA). The bacterial 16S rRNA gene (V3‐V4 region) was amplified using the primer pairs 338F (5″‐ACTCCTACGGGAGGCAGCAG‐3″) and 806R(5″‐GGACTACHVGGGTWTCTAAT‐3″) in an ABI GeneAmp 9700 PCR thermocycler (ABI, CA, USA). Microbiota composition was determined on Illumina MiSeq platform (Illumina, San Diego, USA) following the protocol from Majorbio Bio‐Pharm Technology Co. Ltd. Thereinto, operational taxonomic units (OTUs) were clustered in UPARSE (version 7.1). Then, the taxonomy of the OTU representative sequence was evaluated by using RDP Classifier. All data were finally analyzed on the Majorbio Biocloud platform.

### Statistical Analysis

Statistics were processed and analyzed by using GraphPad Prism 7.0 (GraphPad, USA) and SPSS software version 19.0 (IBM, USA). Thereinto, quantitative data are expressed as mean ± standard deviation or median, whereas categorical data are expressed as numbers (percentages). ANOVA and Mann–Whitney U‐test were used to compare the differences in two or more groups. Finally, *p* < 0.05 was considered statistically significant.

## Conflict of Interest

The authors declare no conflict of interest.

## Supporting information

Supporting Information

## Data Availability

Research data are not shared.
