# Supplementary material for: Colon‐Targeted Adhesive Hydrogel Microsphere for Regulation of Gut Immunity and Flora
Source: Adv Sci (Weinh). 2021 Jul 22;8(18):2101619. doi: 10.1002/advs.202101619 (PMC8456273; doi:10.1002/advs.202101619)
Supplement: Supplementary file 1 — Supporting Information [file ADVS-8-2101619-s001.pdf]

© 2021 Wiley-VCH GmbH

ADVANCED SCIENCE
Open Access

Supporting Information

for *Adv. Sci.*, DOI: 10.1002/advs.202101619

Colon-targeted Adhesive Hydrogel Microsphere for Regulation of Gut Immunity and Flora

Hua Liu, Zhengwei Cai, Fei Wang, Liwen Hong, Lianfu Deng, Jie Zhong, Zhengting Wang*, and Wenguo Cui**

Supporting Information

Colon-targeted Adhesive Hydrogel Microsphere for Regulation of Gut Immunity and Flora

Hua Liu, Zhengwei Cai, Fei Wang, Liwen Hong, Lianfu Deng, Jie Zhong, Zhengting Wang*, and Wenguo Cui**

Dr. H. Liu, Dr. L. Hong, Prof. J. Zhong, Dr. Z. Wang
Department of Gastroenterology, Ruijin Hospital, Shanghai Jiao Tong University School of Medicine, 197 Ruijin 2nd Road, Shanghai 200025, P. R. China.
E-mail addresses: zhengtingwang@shsmu.edu.cn (Z. Wang), jimmyzj64@medmail.com.cn(J. Zhong)

Dr. Z. Cai, Dr. F. Wang, Dr. L. Deng, and Prof. W. Cui
Department of Orthopaedics, Shanghai Key Laboratory for Prevention and Treatment of Bone and Joint Diseases, Shanghai Institute of Traumatology and Orthopaedics, Ruijin Hospital, Shanghai Jiao Tong University School of Medicine, 197 Ruijin 2nd Road, Shanghai 200025, P. R. China.
E-mail addresses: wgcui80@hotmail.com (W. Cui).

Keywords: Hydrogel microsphere; oral administration; colon-targeted drug delivery; colitis; gut microbiota

Supplemental Materials and Methods

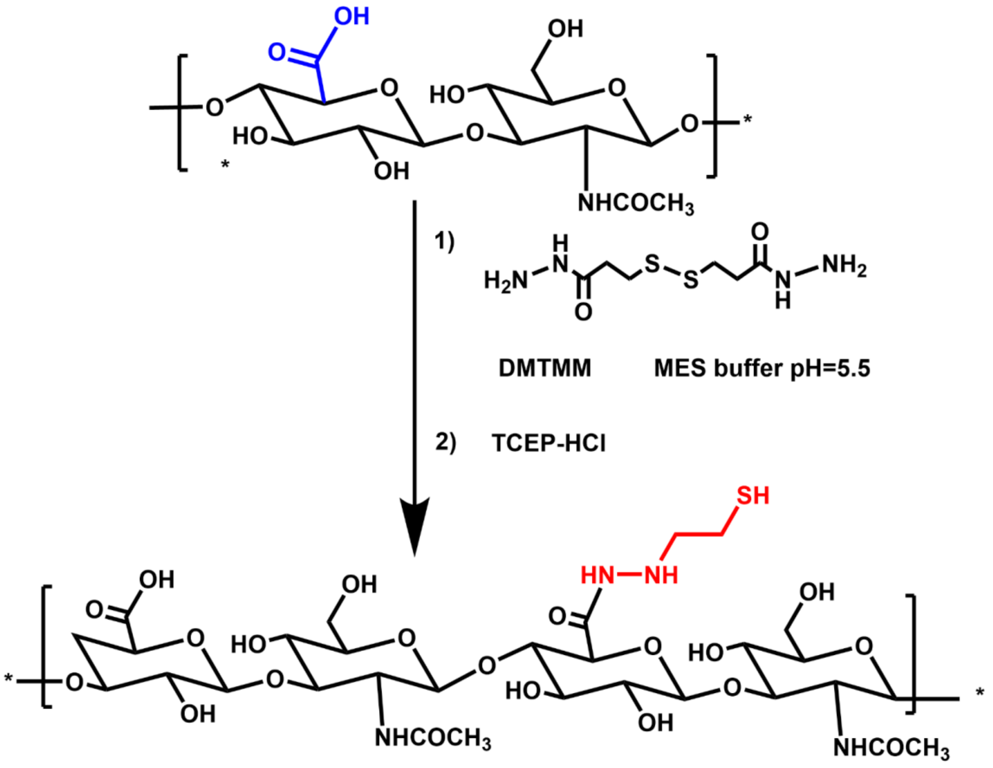

Figure S1. The synthetic route of HA-SH.1) 4-(4,6-Dimethoxy-1,3,5-triazin-2-yl)-4-methylmorpholinium chloride (DMTMM), hyaluronic acid, MES buffer (10.0 mM, pH = 5.5), and RT for overnigth; 2) tris(2-carboxyethyl) phosphine hydrochloride for 2 hours.

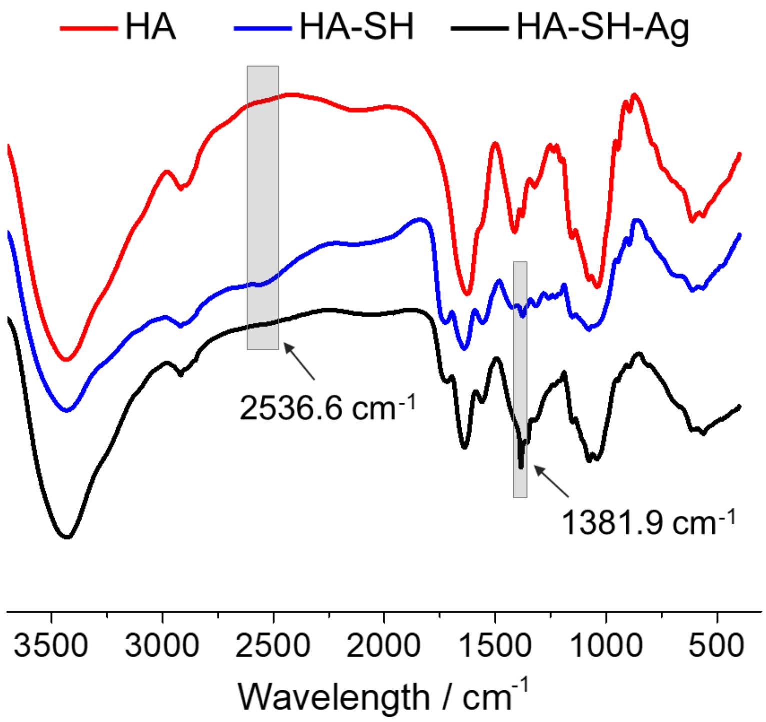

Figure S2. Fourier transform infrared spectrum (attenuated total reflectance) of HA-SH and HA-SH-Ag microsphere. The increased peak at 2536.6 cm^{-1} (-SH flexural vibration) confirmed the grafting of the thiol group. The characterized peak around 1380 cm^{-1} was a large amount of S–Ag coordination bonds that formed in HA-SH-Ag hydrogel between the thiol group of HA and Ag^{+}.

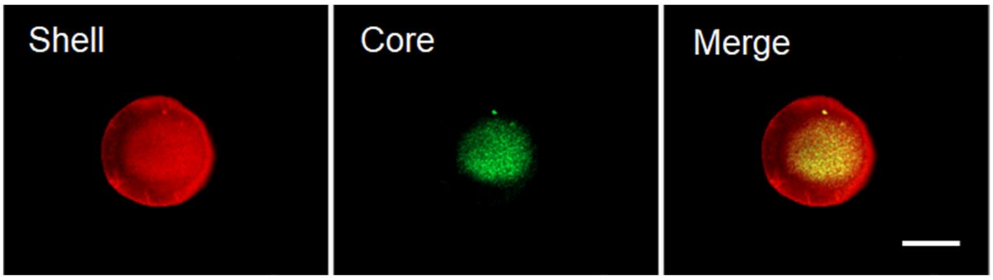

Figure S3. Fluorescence imaging of core(green)-shell(red) structure of HAMs. Scale bar 100 μm.

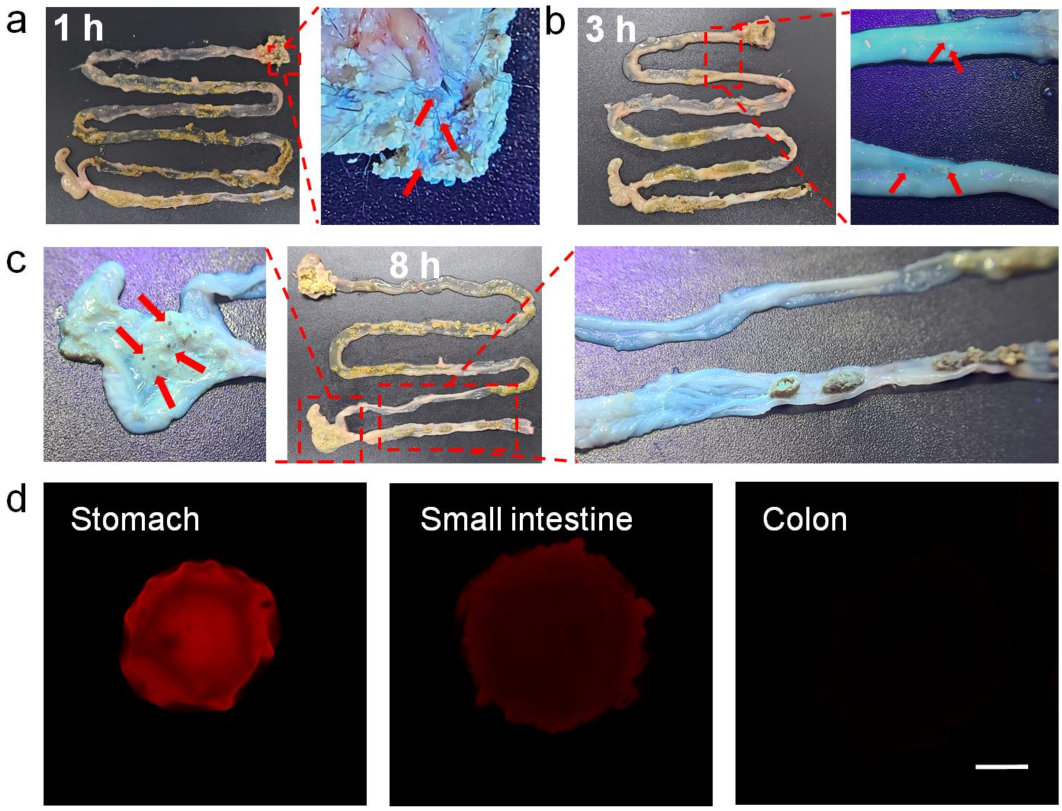

Figure S4. The *in vivo* shell degradation. (a, b, c) Representative image of the digestive tract of mice at the different time points (1hour, 3hours, and 8hours) after oral gavage of HAMs, the tissue with visible HAMs in the intestine were enlarged and HAMs were pointed out (red arrow). (d) HAMs collected from the stomach, small intestine, and colon were observed under the confocal microscope. Scale bar 100 µm.

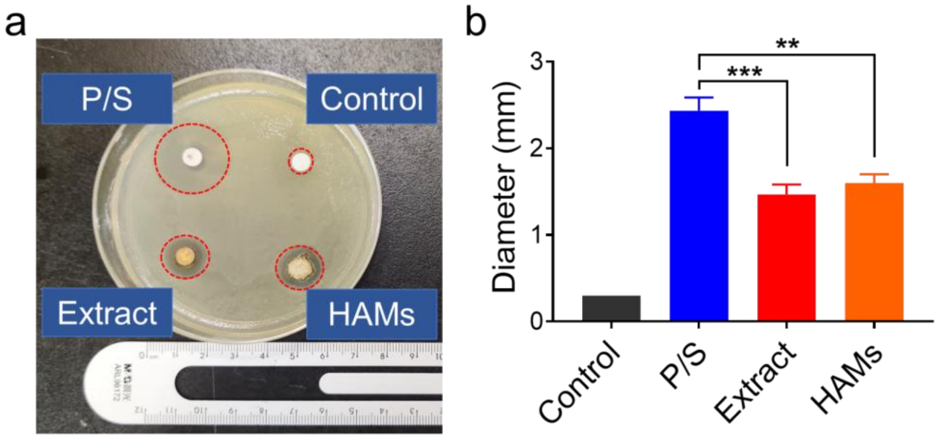

Figure S5. The antibacterial activitywas evaluated by the Kirby-Bauer method. Representative image (a) and diameter analysis (b) of inhibition zones of four groups including penicillin-streptomycin solution, HAMs, and extract of HAMs. The significance between every two groups was assessed by using the Mann-Whitney U-test, ** $P< 0.01$, *** $P< 0.001$. The experiments were repeated 3 times.

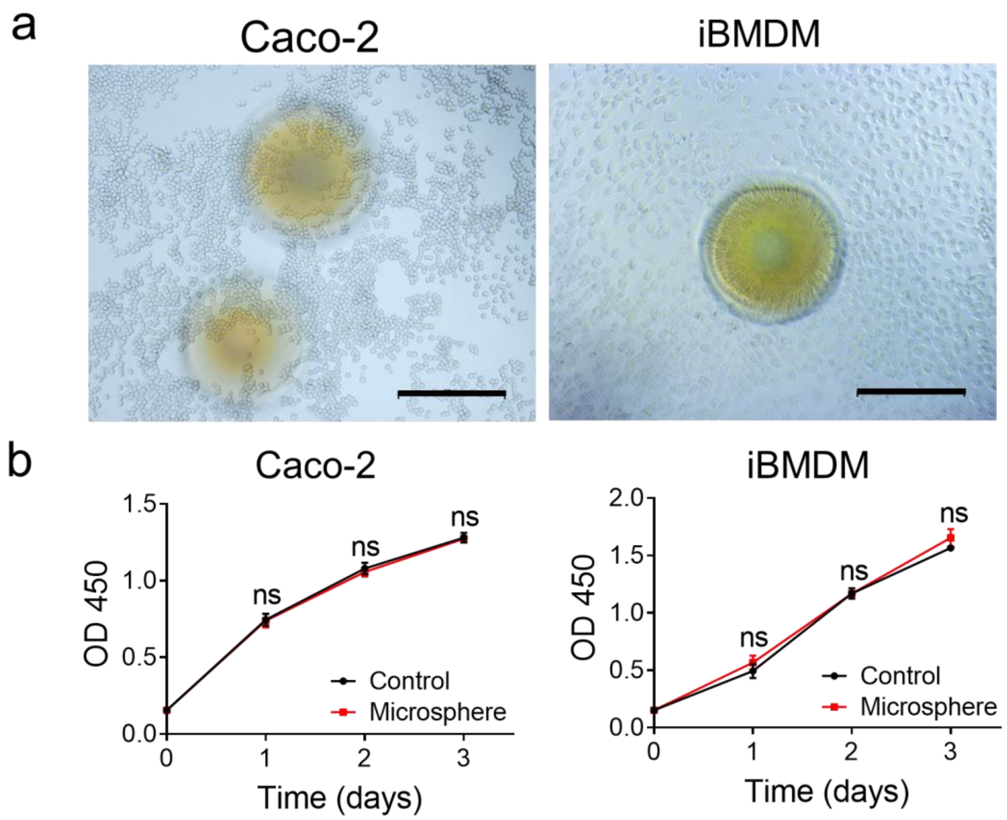

Figure S6. Biocompatibility test.(a) Representative images of cells co-cultured with microspheres. Scale bar 500 μm.(b) The cytotoxicity of HAMs was examined in Caco-2 cell and iBMDM cell by using the cell counting kit-8. The significance between the two groups was assessed by utilizing the Mann-Whitney U-test, ns, not significant.

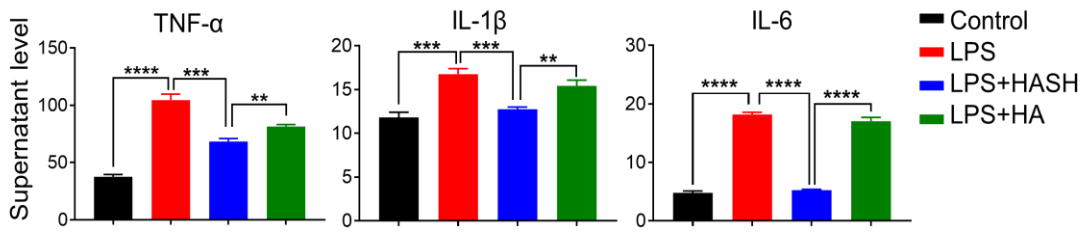

Figure S7. The supernatant concentration (pg/ml) of pro-inflammatory cytokines including interleukin-6, interleukin-1β, and tumor necrosis factor-αwere secreted by iBMDM cells were evaluated by enzyme-linked immunosorbent assay.The experiments were repeated 3 times. The significance between every two groups was assessed by using Mann-Whitney U-test, ns, not significant, ** P< 0.01, *** P< 0.001, **** P< 0.0001.

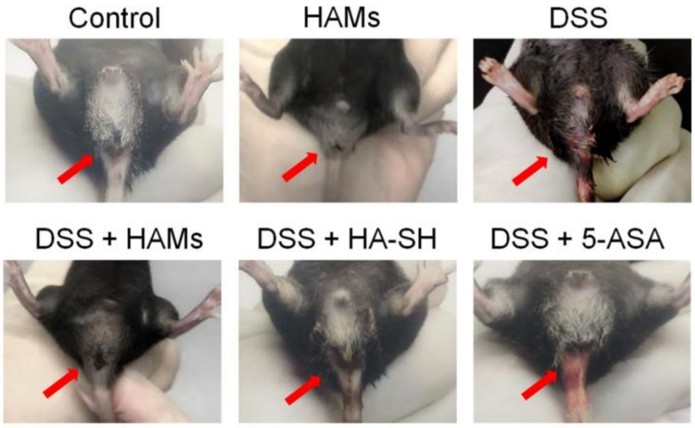

Figure S8. Representative photographs of mice anus shown diarrhea and stool bleeding in different groups.

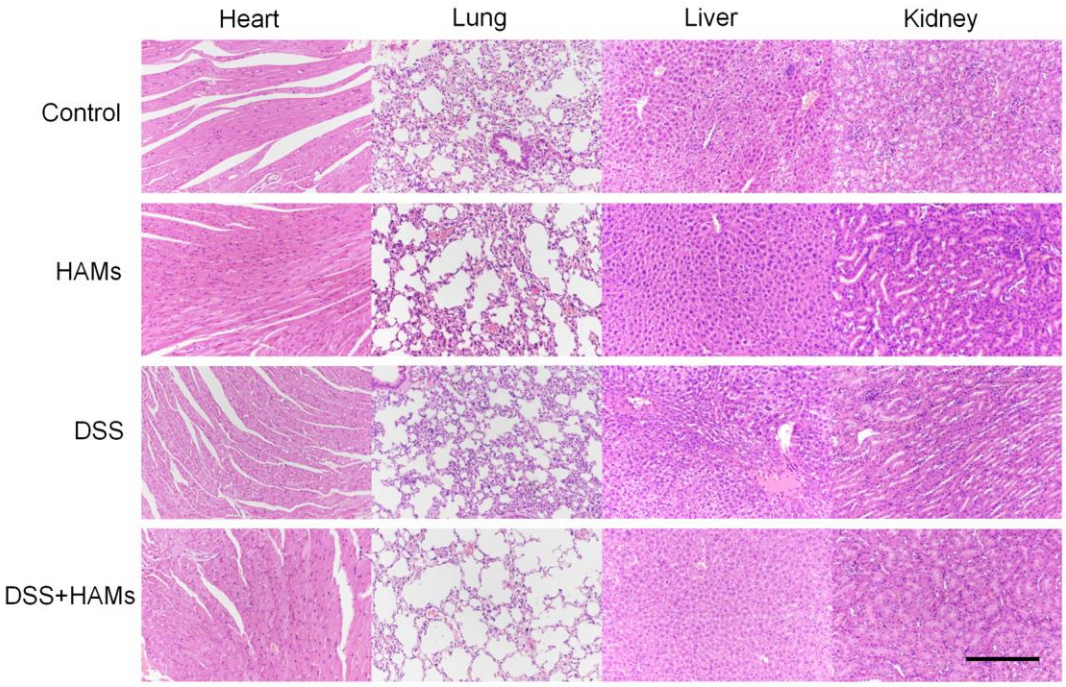

Figure S9. *In vivo* biocompatibility. H&E staining of tissues from the heart, lung, liver, and kidney of each group showed the biocompatibility of HAMs in mice.Scale bar 500 µm.

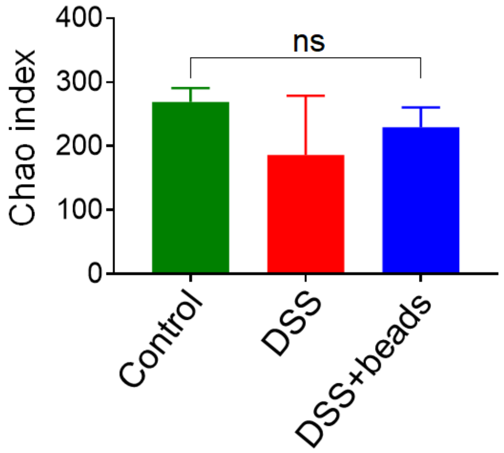

Figure S10. Characteristics of community composition.Chao index of the observed operational taxonomic unit showed the community richness of mice feces between different groups. ns, not significant.
